# Knowledge and Awareness of Compartment Syndrome Among Orthopedic and Emergency Department Nurses: A Multicenter Cross-Sectional Study in Saudi Arabia

**DOI:** 10.7759/cureus.101258

**Published:** 2026-01-10

**Authors:** Hamza M Alrabai, Malek A Albahlol, Abdullah A Alomran, Yazan A Abuhoza, Mohammed K Alghamdi, Mohammad N Khdary, Meshal A Aljudai, Salman z Alotaibi

**Affiliations:** 1 Orthopedics, King Saud University, Riyadh, SAU; 2 College of Medicine, King Saud University, Riyadh, SAU

**Keywords:** acute compartment syndrome, clinical knowledge, emergency nursing, limb ischemia, orthopedic nursing, saudi arabia

## Abstract

Acute compartment syndrome (ACS) is a limb-threatening condition that requires timely recognition and management. Nurses who work in emergency and orthopedic settings play an important role in the early identification and care of patients at risk of developing ACS. This multicenter cross-sectional study assessed the knowledge and awareness of ACS among 400 nurses working in emergency and orthopedic departments across five major hospitals in Saudi Arabia using a validated electronic questionnaire. Overall, 312 (78%) of participants showed moderate to adequate knowledge. Domain-based analysis showed that nurses had higher performance in clinical signs and symptoms and management and prevention, while diagnostic criteria and assessment represented the weakest domains. Higher clinical experience showed a significant association with better knowledge levels (*P* < 0.001), and nurses who had received specialized orthopedic training showed significantly higher knowledge scores compared with untrained nurses (*P* = 0.027). These findings imply that, although overall knowledge scores were adequate, critical deficits in diagnostic assessment suggest that nurses may recognize ACS theoretically but struggle with practical early diagnosis, highlighting the need for targeted, experience-based, and specialty-focused training.

## Introduction

Acute compartment syndrome (ACS) is a limb-threatening surgical emergency caused by increased pressure within the myofascial compartment, leading to compromised blood perfusion and tissue ischemia [1]. In the absence of early diagnosis and appropriate treatment, ACS can result in irreversible muscle and nerve damage, limb amputation, or even death [1]. Fasciotomy is the definitive treatment when performed within the critical window of 4-6 hours [2]. Nurses, particularly those working in orthopedic and emergency departments, play a vital role in the early recognition of ACS, as they are often the first to assess trauma patients. Monitoring changes in pain, neurovascular function, and swelling is of vital importance in raising early clinical suspicion [3].

Despite the critical role of nurses in this regard, several studies have demonstrated inconsistencies in nurses’ knowledge and awareness of ACS. Pokharel [3] reported that only 43.3% of nurses had an adequate understanding of ACS, while Bazezew et al. [4] found moderate to adequate knowledge levels in 61% of surveyed nurses.

Despite the extensive documentation of ACS, there is a lack of research evaluating nurses’ knowledge and awareness of the condition in Saudi Arabia. This study is the first of its kind in Saudi Arabia and aims to assess the knowledge and awareness of ACS among nurses working in orthopedic and emergency departments across multiple hospitals. Additionally, the study explores the relationship between knowledge levels and demographic factors such as education, experience, and specialized orthopedic training.

This study seeks to answer the following question: What is the level of knowledge and awareness of ACS among orthopedic and emergency department nurses in Saudi Arabia? To address this question, a multicenter cross-sectional study was conducted.

Background

Pathophysiology

The prevailing theory regarding the pathophysiology of ACS describes it as tissue ischemia resulting from increased pressure within a closed myofascial compartment. This increase in pressure can be explained by the arteriovenous pressure gradient theory, which suggests that trauma-induced elevation of intracompartmental pressure increases venous pressure, leading to reduced blood flow through the limb. Consequently, the arteriovenous pressure gradient decreases, compromising tissue perfusion and resulting in ischemia [5].

Clinical Diagnosis

The diagnosis of compartment syndrome depends on the recognition of its hallmark symptoms, commonly referred to as the six P’s: pain, poikilothermia, pallor, paresthesia, pulselessness, and paralysis [6]. Early on, there would be a tense feeling in the compartment and pain that is out of proportion [1]. Late signs include paresthesia and paralysis [6].

Fractures are the most common cause of ACS, particularly tibial diaphyseal fractures and distal radial fractures, followed by soft-tissue injury without fracture. Other causes include prolonged static patient positioning during surgical procedures, injection of recreational drugs, limb casting, postoperative bleeding, tourniquet use, lower-limb arthroplasty, and other contributing factors [7].

Management and Complications

Initial nonoperative management of ACS may include relieving splints or compression dressings and elevating the affected limb [8]. Operative interventions, such as fasciotomy, are indicated when conservative measures fail to alleviate compartment pressure [9]. Limb loss or amputation remains among the most severe complications of ACS, particularly following lower-extremity fasciotomy [10]. Fasciotomies are considered time-dependent; a delay of 8 hours or more from the time of injury to treatment is associated with a higher complication rate [2].

## Materials and methods

This cross-sectional study was conducted using an online self-administered questionnaire among registered nurses working in orthopedic and emergency departments across healthcare institutions in Saudi Arabia. Participation was voluntary, and electronic informed consent was obtained before participation. The study was conducted over one year following IRB approval, and nurses from other departments were excluded.

Convenience sampling was used, with recruitment conducted through institutional emails and professional nursing groups, supported by reminder messages to reduce non-response. The participants were anonymous. The sample size was calculated using a single population proportion formula, assuming a 50% prevalence of adequate knowledge, a 95% confidence level, and a 5% margin of error, which resulted in a minimum required sample size of 384 participants. This was increased to 422 to account for non-response, and 400 complete responses were included in the analysis.

Eligible participants were registered nurses with at least six months of experience in orthopedic or emergency care who could read English and provided informed consent. Data were collected using a structured electronic questionnaire adapted from previously published research, with permission from the original author; no changes in the questionnaire were made.

Knowledge was assessed using 22 items, scored as one for correct responses and zero for incorrect responses, with total scores ranging from 0 to 22. Knowledge levels were classified as inadequate (≤13), moderate (14-16), or adequate (≥17).

Data were analyzed using SPSS version 20 (IBM Corp., Armonk, NY). Descriptive statistics were used to summarize participant characteristics and knowledge scores, and binary logistic regression was performed to identify factors associated with knowledge of compartment syndrome prevention. Results were reported as adjusted odds ratios (ORs) with 95% confidence intervals (CIs), and statistical significance was set at *P* < 0.05. Ethical approval and permission to distribute the questionnaire were obtained from KKUH Nursing Research Affairs, and data confidentiality was strictly maintained.

## Results

A total of 400 nurses participated in the study. As shown in Table 1, the majority of participants were male (237, 59.2%) and fell within the age group of 20 to 30 years (172, 43.0%). Regarding education, holders of a Bachelor of Science in Nursing (BSN) constituted the largest group (253, 63.2%), followed by those with an Associate’s degree (84, 21.0%). In terms of professional experience, 172 (43.0%) respondents had between one and five years of experience, while 73 (18.3%) had less than one year. The majority of participants (236, 59%) reported receiving special training in orthopedic nursing. The largest proportion of nurses worked in the Emergency Room (188, 47.0%), followed by the Orthopedic Surgical Ward (106, 26.5%). 

**Table 1:** Demographic and professional characteristics of participating nurses. ADN, Associate Degree in Nursing; BSN, Bachelor of Science in Nursing; MSN, Master of Science in Nursing; DNP, Doctor of Nursing Practice

Variable	Frequency	Percentage
Gender		
Male	237	59.20%
Female	163	40.80%
Age group (years)		
20-30	172	43.00%
30-40	116	29.00%
40-50	57	14.20%
>50	55	13.80%
Education level		
ADN	84	21.00%
BSN	253	63.20%
MSN	35	8.80%
DNP	28	7.00%
Total work experience (in years)		
<1	73	18.30%
1-5	172	43.00%
5-10	65	16.30%
10-15	35	8.80%
15-20	33	8.30%
>20	22	5.50%
Did you get any special training in orthopedic nursing?		
Yes	236	59.00%
No	164	41.00%
Current working area		
Orthopedic Surgical ward	106	26.50%
Orthopedic clinic	56	14.00%
ER	188	47.00%
Others	50	12.50%

Table 2 presents the descriptive analysis of participants' responses to individual questionnaire items. The highest correct response rates were observed for questions related to early manifestations assessment (item 20, 84.3%) and preventive nursing actions during traction (item 18, 334, 83.5%). Conversely, the lowest performance was noted in questions assessing nursing considerations for compartment syndrome assessment (item 11, 57.5%), timing of irreversible muscle damage (item 10, 236, 59.0%), and alternative assessment methods when capillary refill is unavailable (item 15, 235, 58.8%). Within the Clinical Signs and Symptoms domain, while participants demonstrated strong knowledge of the first sign of compartment syndrome (item 8, 314, 78.5%), nearly half (41.0%-42.3%) struggled with questions regarding the timing of muscle damage and assessment considerations.

**Table 2:** Descriptive analysis of participants' responses to knowledge assessment items. n = frequency; % = percentage

Questionnaire item	Correct response, *n* (%)	Incorrect response *n* (%)
Domain 1: Etiology and risk factors		
1. What is traumatic limb compartment syndrome?	256 (64.0%)	144 (36.0%)
2. Compartment syndrome is commonly seen on	285 (71.3%)	115 (28.7%)
3. Which of the following compartments is the most common site for compartment syndrome of the legs?	291 (72.8%)	109 (27.3%)
4. Which of the following are the possible sites for developing compartment syndrome?	312 (78.0%)	88 (22.0%)
5. Which one of the following is the main condition that causes compartment syndrome?	281 (70.3%)	119 (29.8%)
6. Which of the following characteristics of the fascia can cause it to develop compartment syndrome?	270 (67.5%)	130 (32.5%)
Domain 2: Clinical signs and symptoms		
7. What should you recommend for a patient who has compartment syndrome?	254 (63.5%)	146 (36.5%)
8. What is the first sign of compartment syndrome?	314 (78.5%)	86 (21.5%)
9. Which of the following is a late sign of compartment syndrome?	310 (77.5%)	90 (22.5%)
10. In compartment syndrome, with compromised blood supply creating ischemia, irreversible muscle damage occurs within … hours	236 (59.0%)	164 (41.0%)
11. Which of the following nursing considerations is used to assess the compartment syndrome?	231 (57.8%)	169 (42.3%)
Domain 3: Diagnostic and assessment		
12. Which of the following is the diagnostic criterion of the compartment syndrome?	325 (81.3%)	75 (18.8%)
13. One of the common complications of compartment syndrome is	287 (71.8%)	113 (28.2%)
14. A surgical procedure done to relieve pressure in compartment syndrome is	318 (79.5%)	82 (20.5%)
15. When assessing the client's fractured extremity, if the nurse is unable to assess the capillary refill in the beds, what should the nurse do?	235 (58.8%)	165 (41.3%)
16. Which of the following 6 P's are associated with compartment syndrome?	284 (71.0%)	116 (29.0%)
Domain 4: Management and prevention		
17. Which of the following is the main nursing consideration to prevent the development of compartment syndrome?	324 (81.0%)	76 (19.0%)
18. If the patient is on skin traction, what nursing action should be taken to prevent compartment syndrome?	334 (83.5%)	66 (16.5%)
19. After application of a cast in the upper extremities, how should the nurse position the client's limb for the first 24 hours to prevent compartment syndrome?	325 (81.3%)	75 (18.8%)
20. A client who has had a plaster of Paris cast applied to his forearm is receiving pain medication. To detect early manifestations of compartment syndrome, which of the reassessments should the nurse make?	338 (84.5%)	62 (15.5%)
21. While caring for a client with a newly applied plaster of Paris cast, the nurse makes note of all the following conditions. Which assessment finding requires immediate notification of the physician?	277 (69.3%)	123 (30.8%)
22. Which one of the following is the method of assessing for the sign of circulatory impairment in a client with a fractured femur is to ask the client to?	289 (72.3%)	111 (27.8%)

Participants' knowledge was assessed across four thematic domains, as detailed in Table 3. The highest mean knowledge score was observed in the *Management and Prevention* domain, where participants achieved a mean score of 4.61 ± 1.31, representing 76.83% of the total possible score. The domain of *Clinical Signs and Symptoms* followed closely with a mean score of 3.81 ± 1.01 (76.2%). Conversely, the lowest scores were recorded in the *Diagnostic and Assessment* domain, with a mean of 3.29 ± 1.17 (65.8%). The domain covering *Etiology and Risk Factors* yielded a mean score of 4.24 ± 1.39 (70.7%).

**Table 3:** Mean knowledge scores by thematic domain. The mean score (%) was calculated by dividing the mean score by the number of items in each domain. SD, standard deviation

Thematic domain	Number of items	Mean ± SD	Mean score (%)
Etiology and risk factors	6	4.24 ± 1.39	70.7%
Clinical signs and symptoms	5	3.81 ± 1.01	76.2%
Diagnostic and assessment	5	3.29 ± 1.17	65.8%
Management and prevention	6	4.61 ± 1.31	76.8%

Table 4 presents the association between demographic characteristics and knowledge levels using the Pearson Chi-square test. Statistical analysis revealed a significant association between total work experience and knowledge level (χ2 = 48.8, *P* < 0.001). Specifically, nurses with less than one year of experience had the highest rate of inadequate knowledge (32, 43.8%), whereas those with 10 to 15 years of experience demonstrated higher proficiency, with 30 (85.7%) showing moderate to adequate knowledge. Specialized training in orthopedic nursing was also significantly associated with better knowledge levels (χ² = 4.79, *P* = 0.029). Of those with training, 193 (81.8%) achieved moderate to adequate scores, compared with 119 (72.6%) of those without training. Additionally, the current working area showed a significant association with knowledge levels (χ² = 24.0, *P* < 0.001). Participants working in the Emergency Room and Orthopedic Surgical ward demonstrated higher knowledge levels, with 158 (84.0%) and 87 (82.1%) achieving moderate to adequate scores, respectively. Higher rates of inadequate knowledge were observed among nurses in the Other category (21, 42.0%) and Orthopedic clinics (20, 35.7%). Other demographic variables, including age group (χ2 = 4.82, *P* = 0.185), gender (χ2 = 2.27, *P* = 0.131), and level of education (χ2 = 0.369, *P* = 0.947), did not show a statistically significant association with knowledge levels.

**Table 4:** Association between demographic characteristics and knowledge levels of acute compartment syndrome. Test statistics from Pearson's chi-square test; *n* = frequency; % = percentage; χ2 = Pearson Chi-square value; *P* = probability value. Statistical significance set at *P* < 0.05. ADN, Associate Degree in Nursing; BSN, Bachelor of Science in Nursing; MSN, Master of Science in Nursing; DNP, Doctor of Nursing Practice

Characteristics	Inadequate knowledge, *n* (%)	Moderate to adequate knowledge, *n* (%)	*χ*²	*P*-value
Age group				
20-30	31 (18.0%)	141 (82.0%)	4.82	0.185
30-40	26 (22.4%)	90 (77.6%)		
40-50	17 (29.8%)	40 (70.2%)		
50+	14 (25.5%)	41 (74.5%)		
Gender				
Female	42 (25.8%)	121 (74.2%)	2.27	0.131
Male	46 (19.4%)	191 (80.6%)		
Level of Education				
ADN	19 (22.6%)	65 (77.4%)	0.369	0.947
BSN	54 (21.3%)	199 (78.7%)		
MSN	9 (25.7%)	26 (74.3%)		
DNP	6 (21.4%)	22 (78.6%)		
Total Work Experience (Years)				
Less than 1 year	32 (43.8%)	41 (56.2%)	48.8	<0.001
1-5 years	27 (15.7%)	145 (84.3%)		
5-10 years	16 (24.6%)	49 (75.4%)		
10-15 years	5 (14.3%)	30 (85.7%)		
15-20 years	5 (15.2%)	28 (84.8%)		
20+ years	3 (13.6%)	19 (86.4%)		
Special Training (Orthopedic)				
Yes	43 (18.2%)	193 (81.8%)	4.79	0.029
No	45 (27.4%)	119 (72.6%)		
Current working area				
Emergency room	30 (16.0%)	158 (84.0%)	24.0	<0.001
Orthopedic Surgical ward	19 (17.9%)	87 (82.1%)		
Orthopedic clinic	20 (35.7%)	36 (64.3%)		
Other	21 (42.0%)	29 (58.0%)		

**Figure 1:**
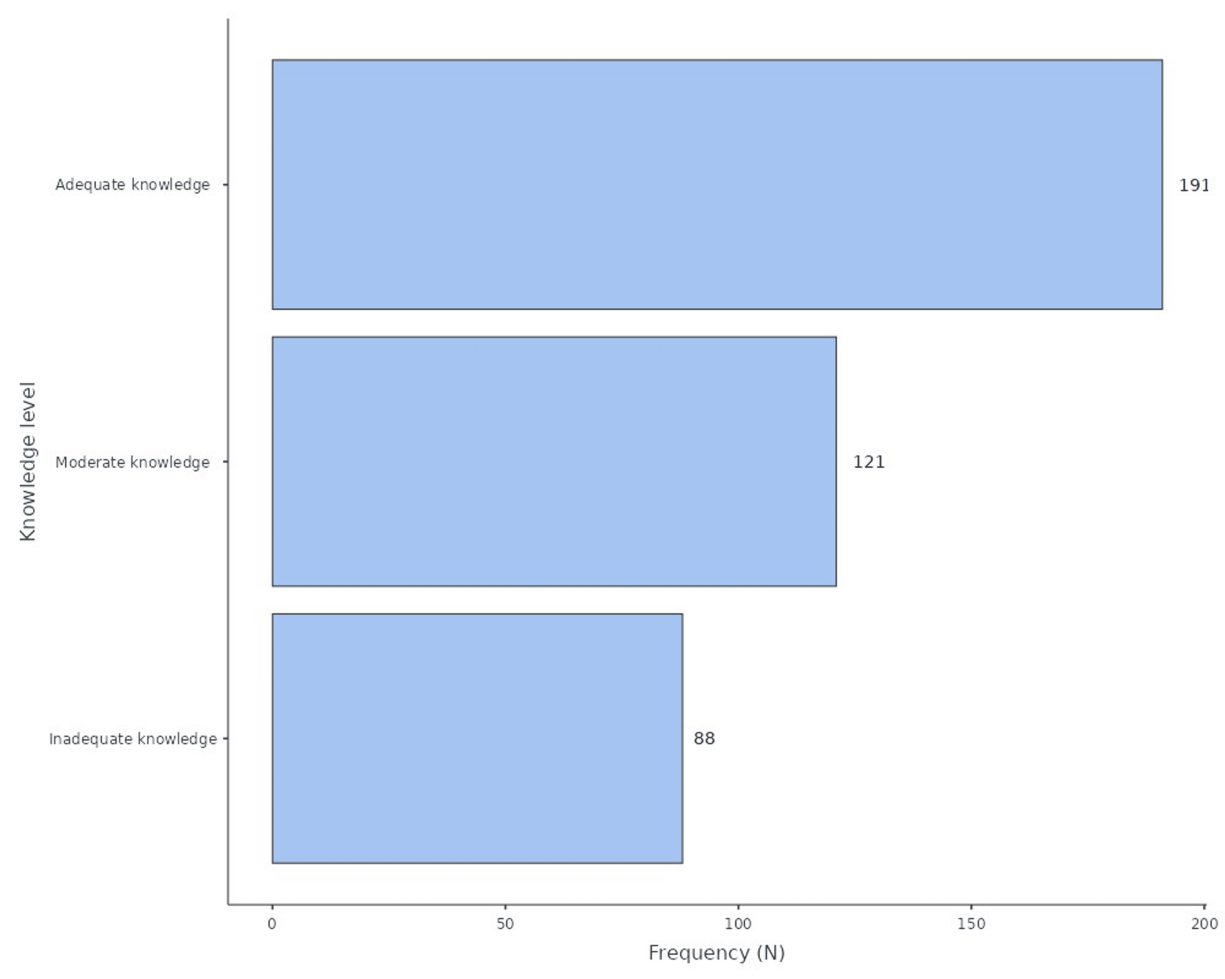
Knowledge levels regarding acute compartment syndrome among participants.

A binomial logistic regression analysis was conducted to identify predictors of moderate to adequate knowledge, as shown in Table 5. Work experience remained a strong predictor. Compared to the reference group (nurses with <1 year of experience), those with 1-5 years of experience were 4.57 times more likely to demonstrate moderate to adequate knowledge (OR: 4.57, 95% CI: 2.39-8.75, *P *< 0.001). The odds of possessing adequate knowledge peaked among nurses with 15-20 years of experience (OR: 8.18, 95% CI: 2.55-26.23, *P *< 0.001). Even those with over 20 years of experience maintained high odds (OR: 7.05, *P *= 0.005). Special orthopedic training remained a significant positive predictor in the regression model. Nurses with this training were nearly twice as likely to have adequate knowledge compared to their untrained counterparts (OR: 1.92, 95% CI: 1.07-3.43, *P *= 0.027). The age group of 40 to 50 years showed a negative association with knowledge scores compared to the 20 to 30 age group (OR: 0.39, 95% CI: 0.17-0.90, *P* = 0.027). Consistent with the previous table, gender and education level were not found to be significant predictors in the multivariate model (Figure 1).

**Table 5:** Binomial logistic regression analysis of factors associated with knowledge levels of acute compartment syndrome. *P*-values are from binomial logistic regression coefficients. Statistical significance set at *P* < 0.05. AOR, adjusted odds ratio; CI, confidence interval; *P* = probability value; ADN, Associate’s Degree in Nursing; BSN, Bachelor of Science in Nursing; MSN, Master of Science in Nursing; DNP, Doctor of Nursing Practice

Predictor	AOR	95% CI (Lower-Upper)	*P*-value
Gender			
Reference Group	1.00	Reference	
Comparison Group	1.46	0.87-2.46	0.151
Work Experience			
<1 year	1.00	Reference	
1-5 years	4.57	2.39-8.75	< 0.001
5-10 years	3.68	1.64-8.26	0.002
10-15 years	6.91	2.18-21.85	0.001
15-20 years	8.18	2.55-26.23	<0.001
20+ years	7.05	1.80-27.63	0.005
Education Level			
ADN	1.00	Reference	
BSN	0.71	0.36-1.41	0.329
MSN	0.92	0.34-2.53	0.877
DNP	0.60	0.19-1.87	0.373
Special Orthopedic Training			
No	1.00	Reference	
Yes	1.92	1.07-3.43	0.027
Age group			
20-30 years	1.00	Reference	
30-40 years	0.61	0.31-1.20	0.154
40-50 years	0.39	0.17-0.90	0.027
50+ years	0.49	0.22-1.08	0.078
Current Working Area			
Orthopedic clinic	1.00	Reference	
ER	2.60	1.16-8.30	0.021
Orthopedic surgical ward	3.81	1.63-8.90	0.002
Other	0.66	0.26-1.70	0.373

## Discussion

This multicenter cross-sectional study evaluated the knowledge and awareness of ACS among orthopedic and emergency department nurses across multiple hospitals in Saudi Arabia. The study population was primarily young male nurses holding a BSN. A significant portion of the participants had between one and five years of clinical experience, and the majority reported having specialized training in orthopedic nursing.

Knowledge and thematic domain performance

In this study, 312 (78.0%) of the surveyed nurses demonstrated moderate to adequate knowledge, which was higher than that reported in other studies. For example, Bazezew et al. in Ethiopia reported that 240 (61.0%) of 410 nurses had adequate knowledge in a multicenter study [4]. Similarly, a Nepalese study conducted at a tertiary hospital in Kathmandu found that 42 (33.9%) had moderate level knowledge and 47 (37.9%) had high level knowledge among 124 nurses [11]. Domain-specific analysis shows that “clinical signs and symptoms” and “management and prevention” had the highest scores, with means of 3.81±1.01 (76.2%) and 4.61±1.31 (76.8%), respectively. This pattern is reassuring as the “clinical signs/symptoms” domain aligns with how bedside monitoring is expected to function in practice. Intractable pain out of proportion is the earliest and most reliable sign; delayed diagnosis can lead to complications or even amputation [5,12,13]. 

In contrast, “diagnostic criteria and assessment” was the weakest domain in the current study, 3.29±1.17 (65.8%), suggesting that nurses can recognize symptoms and follow treatment guidelines, but they may lack proficiency in the detailed clinical judgment required for early diagnosis of ACS. The weakness in “diagnostic criteria and assessment” is consistent with published education audits showing that nurses may disproportionately focus on neurovascular compromise, which is a late feature, rather than prioritizing pain-based early recognition, which can plausibly contribute to delayed escalation [14,15,16]. 

Benefits of specialized training

Specialized orthopedic training was significantly associated with knowledge levels in this study (*P *= 0.029), with 193 (81.8%) of trained nurses achieving moderate to adequate scores, compared with 119 (72.6%) of untrained counterparts. The binomial analysis strengthened this relationship, showing that trained nurses had nearly twice the adjusted odds of possessing adequate knowledge (AOR 1.92, 95% CI: 1.07-3.43, *P *= 0.027). This effect size is remarkably consistent with the Ethiopian findings, in which trained nurses had 1.65 times the odds of good knowledge (95% CI: 1.063-2.562, *P *= 0.026) [4]. The training participation in this study was 236 (59%), and in the Ethiopian study, it was 221 (53.9%). The current study shows a gap in personnel training. In comparison, the Nepalese study presented an even more concerning situation, with 100% of respondents reporting no specific training in trauma management. Supporting this, a UK audit at a district general hospital showed that a simple one-week teaching program dramatically improved nurses’ ability to identify pain out of proportion to injury as the cardinal feature of ACS, with correct responses increasing from 52% to 83% one month after the intervention [14]. 

Work experience effect

Work experience was the strongest factor linked to knowledge in this study (*P *< 0.001). Nurses with 1-5 years of experience were 4.57 times more likely to have adequate knowledge than those with less than 1 year, and this effect increased with more experience, reaching its peak among those with 15-20 years. Even nurses with over 20 years of experience had high odds (OR: 7.05), showing that knowledge builds over time. The Ethiopian study found similar results: nurses with more than 15 years of experience were much more likely to have good knowledge than those with 5 years or less (95% CI: 1.762-10.045, *P *= 0.001) [4]. The Nepalese study, however, found no statistically significant association between professional experience and knowledge level [11].

Limitations

This study has several limitations. The use of convenience sampling via an online questionnaire may limit the generalizability of the findings. The cross-sectional design precludes causal inference. While the sample size was adequate, the results may not be fully representative of all orthopedic and emergency department nurses across Saudi Arabia. In addition, findings may be affected by multicenter variability and self-report bias.

## Conclusions

This multicenter cross-sectional study highlights that orthopedic and emergency department nurses in Saudi Arabia demonstrated an overall acceptable level of knowledge regarding ACS; however, important gaps persist. While nearly half of the participants demonstrated adequate knowledge, deficiencies were identified in pathophysiology, critical intervention timeframes, and diagnostic assessment, which may hinder timely recognition and management.

Clinical experience and specialized orthopedic training were the primary factors associated with higher knowledge levels, emphasizing the importance of practical exposure and focused training over academic qualification alone. These findings support the need for structured, targeted educational programs emphasizing early diagnosis and initial management strategies to enhance clinical preparedness. Addressing these gaps may improve patient outcomes and reduce preventable complications such as limb loss and permanent neurological deficits.
